# Impact of *Trichoderma afroharzianum* infection on fresh matter content and grain quality in maize

**DOI:** 10.3389/fpls.2024.1436201

**Published:** 2024-07-23

**Authors:** Annette Pfordt, Lara Änne Steffens, Tom Raz, Marcel Naumann

**Affiliations:** ^1^ Institut of Plant Pathology and Crop Protection, Department of Crop Sciences, University of Goettingen, Goettingen, Germany; ^2^ Institut of Plant Nutrition and Plant Physiology, Department of Crop Sciences, University of Goettingen, Goettingen, Germany

**Keywords:** *Trichoderma afroharzianum*, maize, ear rot, amylase activity, quality

## Abstract

*Trichoderma afroharzianum*, a ubiquitous soil-borne fungus found on plant roots and decaying residues, displays competitive traits and mycoparasitic behavior against diverse microorganisms. Selected strains of this fungus are known in agriculture for their beneficial effects on plant growth and as bio-fungicides. However, recent findings have pinpointed *Trichoderma afroharzianum* as the causal agent behind maize ear rot disease in Europe since 2018, notably impacting maize cobs in Germany, France, and Italy. This study aims to evaluate the severity of *Trichoderma* ear rot disease on maize fresh matter content and specific quality parameters under semi-field conditions. Two distinct maize varieties were artificially inoculated with a pathogenic Trichoderma isolate at the flowering stage using needle pin or silk channel methods. Disease severity was assessed visually at the time of harvest based on the percentage of infected kernels according to EPPO Guidelines (PP 1/285). Fresh matter content and quality parameters such as alpha-amylase activity, C/N ratio, water, and sugar content were analyzed. Results showed that needle pin inoculation led to higher disease severity (60%) compared to silk channel inoculation (39%). Cob weight decreased significantly at the highest disease severity level by up to 50% compared to control plants. In both varieties, alpha-amylase activity increased significantly with higher *Trichoderma* disease severity, resulting in starch degradation and increased glucose release. The germination rate was severely affected by the infection, with only 22% of grains germinating, and the seedlings showed shortened and deformed growth. This is the first report on *Trichoderma* ear rot infection and its effect on fresh matter content and quality parameters in maize after artificial inoculation under field conditions. The results address an important knowledge gap and provide valuable insights into the infection pathway and impact on maize quality.

## 1 Introduction

Maize (*Zea mays*) is an important staple food consumed by millions of people worldwide. According to the Food and Agriculture Organization of the United Nations (FAO), the worldwide production of maize in 2021 was estimated at 1.189 billion tons on about 203 million hectares (FAOSTAT, 2021). It is a crucial crop providing energy and nutrients, particularly carbohydrates, fiber, vitamins, and minerals for human consumption (Rouf Shah et al., 2016; Chen et al., 2021). In addition, maize plays an essential role in animal feed, especially for poultry, swine, and cattle, due to its high energy content, moderate protein content, and good digestibility (Lütke Entrup et al., 2013). However, maize is susceptible to several diseases, including viral, fungal, and bacterial infections, which can cause significant yield losses and quality impairments. Particularly, ear rot diseases can produce mycotoxins such as trichothecene, fumonisins, aflatoxins, and deoxynivalenol, which pose serious health risks to humans and animals (Bottalico, 1998; Cotten and Munkvold, 1998; Munkvold and White, 2016). Some *Trichoderma* species are also known to produce cytotoxic and immunosuppressive trichothecenes like trichodermin, trichodermol, and harzianum A, causing a range of health issues including inhibition of protein synthesis, alterations in membrane structure leading to increased lipid peroxidation, and inhibition of electron transport activity in the mitochondria (Sharma et al., 2016; Cope, 2018).

Members of the genus Trichoderma are opportunistic fungi capable of rapidly colonizing diverse niches in different environments (Druzhinina and Kubicek, 2017; Sun et al., 2019; Gao et al., 2020). Various *Trichoderma* spp. have been extensively studied and are used in crop protection as biocontrol agents against plant pathogens. They promote plant health by either directly controlling pathogens through mycoparasitism, competitive inhibition, and antibiosis, or indirectly by promoting plant growth, stimulating systemic plant defenses, and enhancing rhizosphere competence (Sood et al., 2020; Ferreira and Musumeci, 2021). *Trichoderma* spp. are known to produce a variety of enzymes, including chitinases, glucanases, proteases, and cellulases, which can help degrade the cell walls of plant pathogens and prevent their growth (Schuster and Schmoll, 2010). Moreover, *Trichoderma* spp. are used in industrial processes because of their ability to produce and secrete enzymes such as amylase, which are commercially applied in food processing, biofuel production, and the textile industry (Mach and Zeilinger, 2003).

Despite their widely considered beneficial roles, certain members of this fungal genus have been reported to be pathogenic, causing severe damage in maize (Haslam, 1910; Sutton, 1972; Pfordt et al., 2020; Chen et al., 2023). The earliest report of a *Trichoderma* species as a maize pathogen was in Haslam’s 1910 master’s thesis from the University of Kansas, which described *T. lignorium* as a greenish-yellow wet mold growing between maize kernels. This infection often led to grain germination on the cob and was reported widely in Riley County (Kansas, USA) (Haslam, 1910). In 1972, Sutton isolated *T. koningii* from stunted maize plants in southern Ontario. Three years later, McFadden and Sutton (1975) reported *T. koningii*, *T. harzianum*, and *T. hamantum* as the causes of first internode lesions in maize. The first mention of Trichoderma associated with maize ear rot was published by the University of Illinois, Department of Crop Sciences in 1991 identifying *T. viride* as the causal agent of ear infections after mechanical damage by insects. Similar findings were later reported in farm management reports from Iowa (Wise et al., 2016), Ohio (The Ohio State University, 2004), and Kentucky (Vincelli, 2014). In 2020, the disease was reported for the first time in Europe, identifying *T. afroharzianum* as the causal agent of maize ear rot in southern Germany (Pfordt et al., 2020). The disease is characterized by massive mycelial growth along with the production of green conidia in the inter-kernel regions and on the outer surface of the husk. Infected cobs appear small with soft rot symptoms and premature germination of the kernels inside the husk leaves. Besides visible symptoms, infections lead to a reduction in dry and fresh matter content after artificial inoculation in the greenhouse (Pfordt et al., 2020; Sanna et al., 2022). Since 2020, the disease has been described in Germany, France, and Italy, primarily after dry and hot growing seasons (Pfordt et al., 2020; Sanna et al., 2022).

The objective of this study was to investigate the effects of Trichoderma ear rot infection on the fresh matter content and certain quality parameters like alpha-amylase activity, water, and sugar content, C/N ratio, and germination rate of maize after artificial inoculation under field conditions. Artificial inoculation was conducted using two distinct inoculation methods, silk channel inoculation, and needle pin inoculation. Additionally, we aim to identify the reasons for premature germination of kernels within the husk, as this contributes to yield loss and overall crop quality. Since there is currently very little known about Trichoderma ear rot, this study provides interesting insights into the infection pathway. By gaining a better understanding of the impact of Trichoderma ear rot on fresh matter content and quality parameters, more effective strategies can be developed to control and prevent this infection.

## 2 Materials and methods

### 2.1 Fungal cultivation and inoculum preparation

*T. afroharzianum* strains were originally isolated from maize cobs and stalks collected from naturally infected silage and grain maize in Germany and France in 2018 and 2019 (Pfordt et al., 2020). Spore suspensions containing isolates from three *T. afroharzianum* strains, namely AP18TRI1 (Croix de Pardies, France), AP18TRI2 (Künzing, Germany), and AP18TRI3 (Pocking, Germany) (**Supplementary Table 1**), were individually produced and subsequently mixed in equal proportions to form a composite isolate (TriMix). To cultivate the isolates, single-spore cultures were transferred onto potato dextrose agar (PDA) plates and then incubated at 23°C in the dark in a growth chamber (Mytron, Heiligenstadt, Germany). PDA plates were supplemented with two antibiotics: 400 µg/ml streptomycin (Duchefa Biochemie, Haarlem, Netherlands) and 30 µg/ml rifampicin (AppliChem, Darmstadt, Germany). After a two-week incubation period, sterile water was added to the plates, and the conidia were harvested by scraping with a microscope slide. The resulting conidial suspension was filtered through gauze, and the cell density was adjusted to 1x10^6^ conidia per ml using a Thoma hemocytometer (Merck, Darmstadt, Germany).

### 2.2 Plant cultivation and inoculation procedure

The trial was conducted under open field conditions at the Department of Plant Pathology and Plant Protection, Faculty of Crop Sciences, Georg-August University, Göttingen, Germany. Six individual plots, each measuring 180 cm x 180 cm, were used for the study. The plots were arranged as a split-plot design in a randomized complete block design with three replications and two different maize varieties: ‘LIKEit’ (Deutsche Saatveredelung AG) and ‘Mallory’ (Lima Grain). Fertilizer was applied before sowing according to N_min_ analysis at a rate of 159 kg/ha.

Within each plot, three rows of 16 plants per row were planted at a standard spacing of 10 cm (plant spacing) and 75 cm (row spacing). Each row was treated separately; one row was inoculated with *T. afroharzianum* via the silk channel while another row was inoculated with *T. afroharzianum* via needle pin. To establish control conditions, half of the plants in a row were inoculated with water via the silk channel and the other half of the same row received a similar treatment with a needle pin (**Supplementary Figure 1**). Inoculation was carried out ten days after full flowering (BBCH 65) using the aforementioned methods. Silk channel inoculation entailed the injection of 2 ml of either spore suspension or water into the silk channel above the tip of the maize cob using a sterile syringe. Needle pin inoculation was performed by inserting a pin bar with four pins attached to a plastic handle, previously dipped in the suspension or water, into the center of the maize cob.

### 2.3 Visual disease assessment and sample preparation

Visual disease severity was assessed as percentage of infection of every cob (n=288) four weeks post-inoculation at BBCH 80 according to EPPO guidelines (PP1/285) (EPPO Bulletin, 2015). Subsequently, cobs were harvested and categorized into five distinct disease severity classes per variety, with four replications based on their infection levels: K0 (control), K1 (0% disease severity), K2 (1-30% disease severity), K3 (31-70% disease severity), and K4 (71-100% disease severity). Upon completion of the visual assessment, maize cobs were weighed, and the kernels were meticulously separated from the cob. Subsequently, both the kernels and rachis were individually ground and subjected to freeze-drying for 48 hours at -80°C (GOT2000, Mitsubishi; VaCo 5, Zirbus). It’s important to note that the term ‘maize cob’ always refers to a combination of the analysis results obtained from both the kernels and rachis.

### 2.4 Weather monitoring

Weather conditions, including temperature and precipitation, were monitored throughout the growing season using mobile weather stations set up near the field trials. Rainfall was recorded using rain gauges in the fields to provide valuable data for understanding the effects of weather on the development and progression of *Trichoderma* ear rot in maize crops.

### 2.5 Water content analysis and calculation

For water content analyses, 80 maize plants of the ‘LIKEit’ variety were cultivated in the greenhouse using a soil mixture consisting of potting soil, compost, and sand in a 2:2:1 ratio. The pots were arranged in a randomized complete block design with three replications and placed in the greenhouse at 25°C. Irrigation was carried out as needed. Forty plants were inoculated with the TriMix isolate using each of the inoculation methods (silk channel and needle pin) as described above, involving inoculation with 1 ml of the spore suspension at full flowering (BBCH 65). Twenty plants for each inoculation method were inoculated with water as a control.

Four weeks after inoculation, disease severity was assessed as mentioned above and cobs were weighed to quantify its fresh weight (FW). Afterword’s, cobs were dried at 65°C for 48 hours and reweighed to determine their dry weight (DW). The water content was calculated as the difference between the FW and DW.

### 2.6 Sugar content quantification

To quantify soluble sugars (glucose, fructose, and sucrose), high-performance liquid chromatography (HPLC) was employed following the protocol outlined by Koch et al. (2019), with minor adaptations for analyzing kernels and rachis samples. Initially, 0.75 g of the freeze-dried and milled sample material was placed into 15 ml centrifuge tubes and resuspended with 3 ml of distilled water. The mixture was homogenized by horizontally shaking for one hour. To precipitate proteins, 0.5 ml of Carrez I solution (3.6 g K_4_Fe(CN)_6_ in 100 ml distilled water) and 0.5 ml of Carrez II solution (7.2 g H_14_O_11_SZn in 100 ml distilled water) were added to the sample solution. The solution was then centrifuged at 5544 g at room temperature for 20 min using a Megafuge™ 16 (Thermo Fisher Scientific™, Waltham, Massachusetts, USA). After removing the supernatant, it was transferred to a 10 ml flask. The remaining pellet was dissolved again in 3 ml of distilled water, shaken horizontally for 1 hour, and centrifuged for 20 min at 5544 g. This process was repeated twice to ensure thorough dissolution and separation of the sample components. After combining the supernatants from the first, second, and third centrifugation steps, the flasks were filled up to 10 ml with distilled water. The samples were then filtered using filter paper (Type 615 ¼, Macherey-Nagel, Düren, Germany), and approximately 2 ml of the extracts were stored in screw-cap vials at -20°C until further analysis. Prior to HPLC analysis, the samples were thawed and centrifuged again at room temperature at 12,000 rpm for 30 min using the Megafuge™ 16 (Thermo Fisher Scientific™, Waltham, Massachusetts, USA). The supernatant was filtered using a 13 mm syringe filter holder (VWR International, Radnor, PA), and the extracts were quantified via HPLC analysis (Jasco, Pfungstadt, Germany). The analysis utilized an injection volume of 20 µl with acetonitrile as the eluent, flowing at a rate of 1 ml min^-1^, and maintaining a column temperature of 22°C. Measurement was carried out using a refractive index detector.

### 2.7 Determination of alpha-amylase activity

To determine alpha-amylase activity, the α-amylase assay kit (Ceralpha method) from Megazyme (Bray, Ireland) was utilized following the manufacturer’s instructions, summarized below. Initially, 3 g of freeze-dried and milled material of kernels and rachis were weighed and mixed with 20 ml of extraction buffer. This buffer consisted of 50 mM sodium malate, 50 mM sodium chloride, 2 mM calcium chloride, and 0.005% sodium azide, adjusted to a pH of 5.4. The mixture was then shaken in a water bath at 40°C for 15-20 min. Subsequently, the solution was filtered through filter paper (Type 615 ¼, Macherey-Nagel, Düren, Germany) into 50 ml centrifuge tubes and incubated at 40°C for 5 min. Meanwhile, 200 µl of high purity α-amylase reagent, containing 54.5 mg of p-nitrophenyl α-D-maltoheptaoside (blocked) and 125 U of a thermostable α-glucosidase dissolved in 10 ml distilled water, was incubated at 40°C for 5 min. Following this, 200 µl of the filtered extracts were added to 200 µl of high purity α-amylase reagent. For the blanks, 200 µl of extraction buffer was added instead of the filtered extracts. After 20 min, 3 ml of stopping reagent, consisting of 1% [w/v] tri-sodium phosphate solution, was added, and the tube contents were vortexed vigorously. Finally, the absorbance of the solution was measured at 400 nm using a BioTek Synergy HTX Multimode Reader (Agilent Technologies, Santa Clara, United States).

### 2.8 Determination of germination rate and plant length

Fifty seeds from TriMix-inoculated cobs and fifty seeds of water-inoculated cobs were randomly selected and subjected to surface disinfection in a 0.25% silver nitrate (AgNO_3_) solution for 10 min. All seeds were sown in a soil mixture comprising potting soil, compost, and sand in a ratio of 2:2:1 and arranged in a completely randomized design with two replications per treatment in the greenhouse. Plants were maintained at a temperature of 25°C and were irrigated as necessary. The number of germinated seeds was recorded, and the shoot length was measured at BBCH 32.

### 2.9 Quantification of C/N ratio

Assessing soil and plant material using a commercial automated CHN elemental analyzer is now standard practice. Numerous studies (McGeehan and Naylor, 1988; Jimenez and Ladha, 1993) have demonstrated a reliable correlation between established methods for determining total nitrogen (N), such as the wet-oxidation Kjeldahl method (Kjeldahl, 1883) or the Walkley-Black method (Walkley and Black, 1934) for soil carbon (C) analysis. The C/N ratio was assessed through elemental analysis, which involved measuring the total carbon (C) and nitrogen (N) content of the plant material being studied. This analytical procedure included total combustion within a continuous flow of ultra-pure helium, followed by gas analysis using thermal conductivity. For this determination, 3.5-4 mg of lyophilized kernels and rachis material were analyzed, following the methodology outlined by (Chea et al., 2021). Acetanilide, serving as a calibration standard for elemental analysis, was employed in a CN analyzer (NA 1500 N Analyzer, Fison Instruments Ltd, Scotland) to ascertain the C/N ratio of the kernel and rachis samples. The combustion temperature was set at 1200°C, with helium and pure oxygen used as the carrier gases for combustion.

### 2.10 Statistical analysis

Statistical analysis was performed using STATISTICA version 13 (Statistica GmbH, Germany). Experiments were conducted in a fully randomized split-plot design within a randomized complete block design. Variance analysis of mean disease severity was conducted using Box-Cox transformations to achieve variance homogeneity and normal distribution. Multi-factor analysis of variance followed by a Tukey *post hoc* test was performed with a significance level of 5% (p ≤ 0.05). For quality parameters, including sugar content, water content, C/N ratio, alpha-amylase activity, fresh matter content, germination rate, and seedling length, a two-factor analysis of variance (ANOVA) was conducted. Tukey tests were performed with a significance level of 5% (p ≤ 0.05). **Supplementary Material** detailing significant observations and interactions is provided for comprehensive insights into the analyses.

## 3 Results

### 3.1 Weather data

During the semi-field trial, mean air temperature and precipitation were recorded at the field side (**Table 1**). The mean temperature during the vegetation period in Goettingen was +2°C to +2.3°C above the long-term average, while precipitation remained well below the long-term average (DWD, 1971-2020).

**Table 1:** Mean air temperature and precipitation at the field side in Goettingen compared to long-term temperature and precipitation trends (1971-2020) in Goettingen.

	Mean air temperature 2022	Long-term temperature 1971-2020*	Precipitation 2022	Long-term precipitation 1971-2020*
April	8°C	8.1°C	46.4 mm	43 mm
May	14°C	12.9°C	27.2 mm	58 mm
June	18°C	15.5°C	27.8 mm	74 mm
July	19°C	17.4°C	24.2 mm	60 mm
August	20°C	17.3°C	31.6 mm	55 mm

*German weather service 1971-2020.

### 3.2 Visual disease assessment

**Figure 1:**
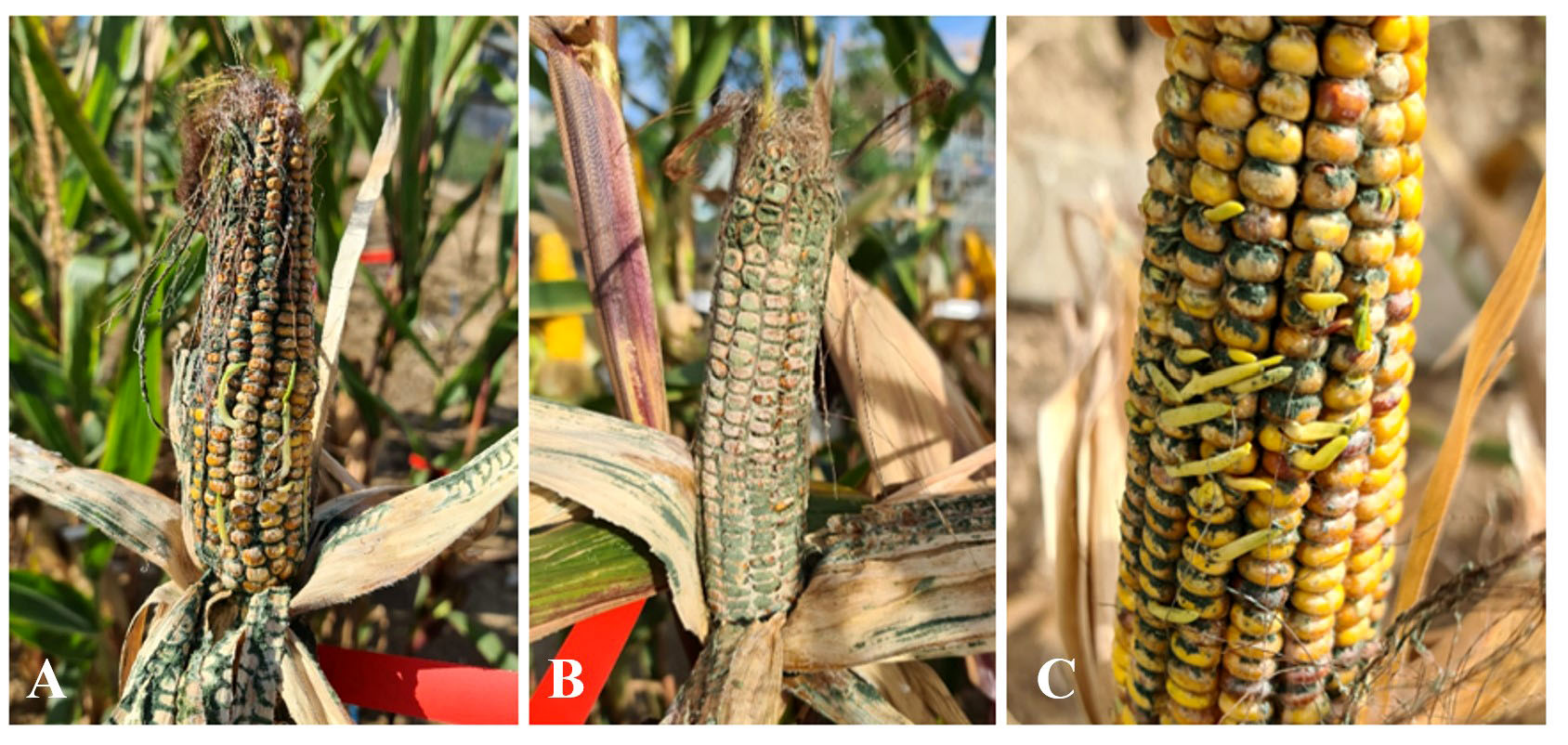
Disease symptoms (100% disease severity) of *Trichoderma* ear rot infection after artificial inoculation under field conditions on Mallory. **(A, B)** Massive production of greenish spores on husk leaves and around the kernels after artificial inoculation; **(C)** early germination of infected kernels.

Four weeks after inoculation, disease assessment and sampling revealed severe symptoms of *Trichoderma* ear rot infection, including mycelial growth with green spores on the kernels and the outer husk leaves (**Figure 1**). Additionally, affected crops exhibited softness due to excessive moisture. Furthermore, several highly infected cobs displayed premature kernel germination inside the husk leaves. To evaluate maize cob susceptibility, they were classified into five distinct disease severity categories per variety, each replicated four times according to their infection levels: K0 (control), K1 (0% disease severity), K2 (1-30% disease severity), K3 (31-70% disease severity), and K4 (71-100% disease severity).

Disease severity was significantly influenced (p ≤ 0.001) by the isolate. Inoculation with TriMix resulted in higher infections (49%) compared to water-inoculated control cobs (3%). Moreover, notable significant differences (p ≤ 0.05) were observed among varieties and inoculation methods, as well as their interactions (**Supplementary Table 2**). LIKEit exhibited significantly lower disease severity (37%) following TriMix inoculation compared to Mallory, which had a mean disease severity of 72% (**Table 2**). Additionally, needle pin inoculation resulted in a higher mean disease severity (60%) compared to silk channel inoculation (39%).

**Table 2:** Disease severity (%) and Standard error (SE) of LIKEit and Mallory after needle pin and silk channel inoculation with TriMix and water control.

Treatment	Variable	Isolate	Disease severity [%] ± SE
Variety	LIKEit	TriMix	36.5 ± 4.5 a
		Control	0.5 ± 0.3 b
	Mallory	TriMix	71.9 ± 23.5 A
		Control	7.4 ± 5.1 B
Inoculation-method	Needle pin	TriMix	59.9 ± 5.5 a
		Control	7.4 ± 4.7 b
	Silk channel	TriMix	39.3 ± 16.1 A
		Control	0.0 ± 0.0 B

Different letters indicated significant differences between classes (p ≤ 0.05, Tukey test).

### 3.3 Fresh matter content

**Figure 2:**
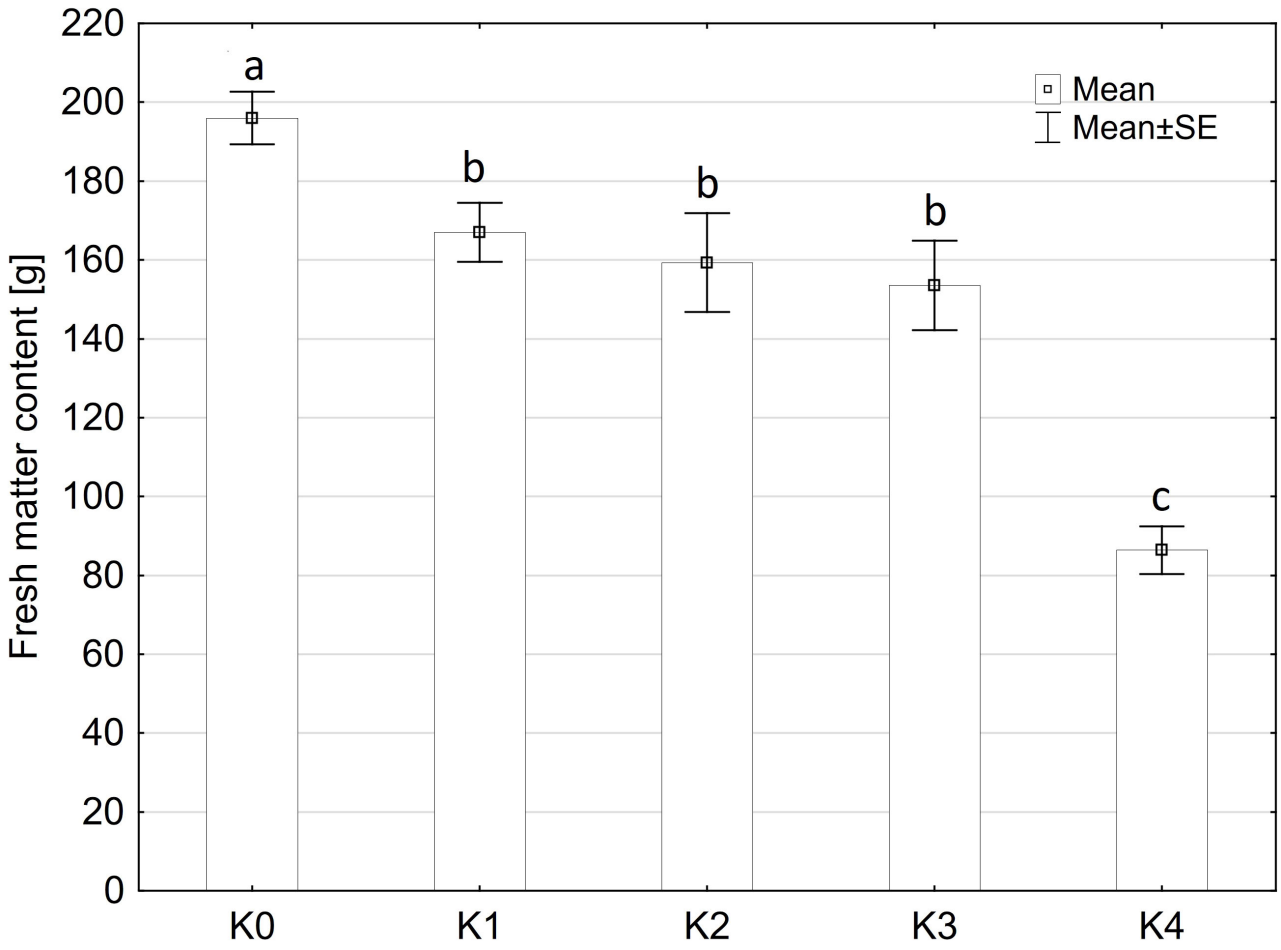
Fresh matter content [%] of maize cobs from both varieties (LIKEit, Mallory) after artificial inoculation with *T. afroharzianum*, at different disease severity classes K0 (control), K1 (0% disease severity), K2 (1-30% disease severity), K3 (31-70% disease severity), and K4 (71-100% disease severity). Error bars represent standard error. Different letters indicate significant differences between classes (p ≤ 0.05, Tukey-Test). n = 288.

The fresh matter content was only significantly influenced by the TriMix inoculation (p ≤ 0.001), compared to water inoculation (**Figure 2**). Neither the main factors variety and inoculation methods nor their interaction had a significant influence on fresh matter content (**Supplementary Table 2**). Fresh matter content of water-inoculated control plants (K0) of both varieties averaged 196 g. However, even at 0% visual disease severity, cob weight decreased significantly to 168 g for both varieties. Fresh matter content was lowest at the highest disease severity level (K4, 71-100% disease severity) with a decrease of 55% to 88.5 g.

### 3.4 Water content

**Figure 3:**
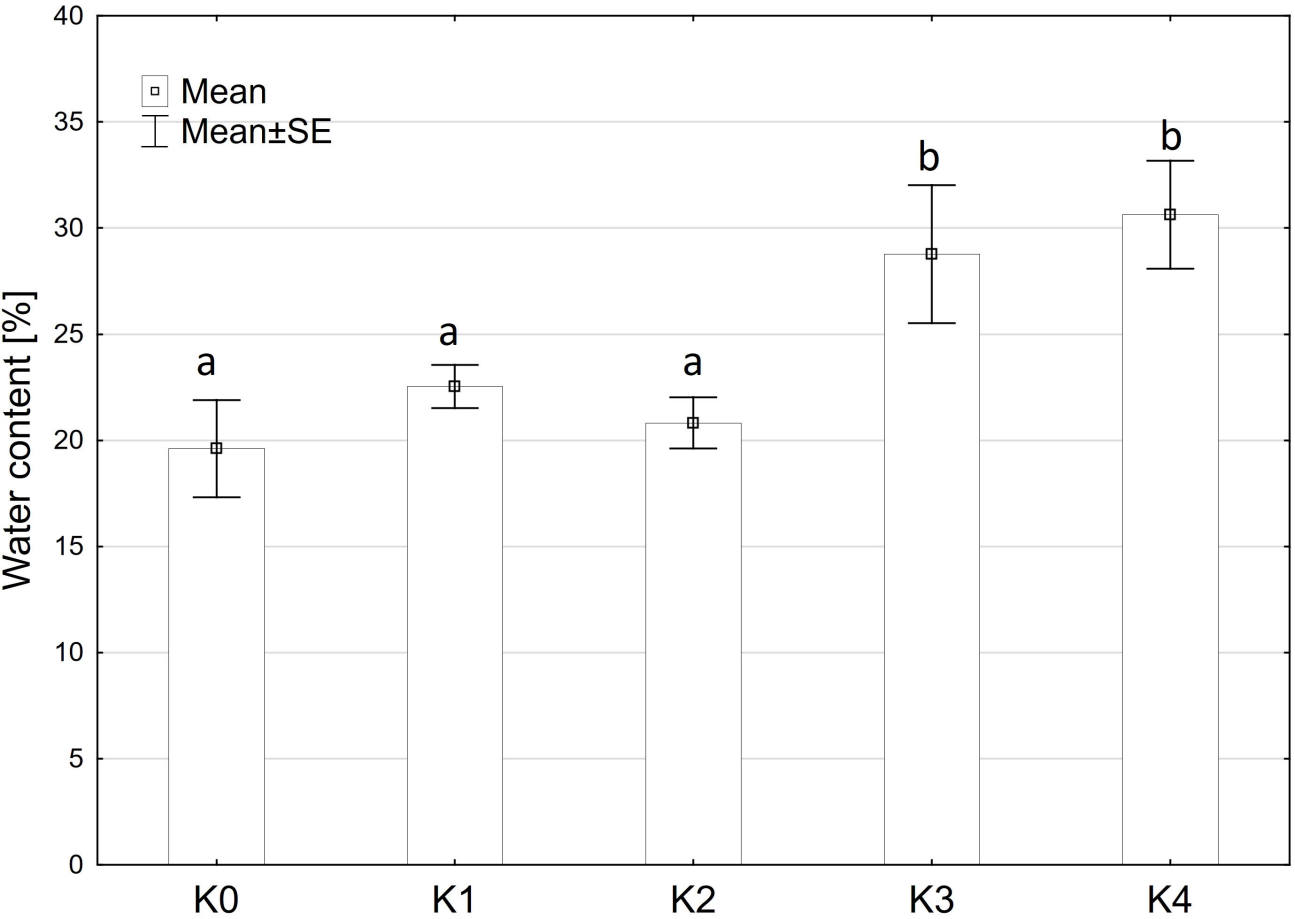
Water content [%] of maize cobs from both varieties (Mallory, LIKEit) after artificial inoculation with *T. afroharzianum* at different disease severity classes K0 (control), K1 (0% disease severity), K2 (1-30% disease severity), K3 (31-70% disease severity), and K4 (71-100% disease severity). Error bars represent standard error. Different letters indicate significant differences between classes (p ≤ 0.05, Tukey-Test). N =288.

The analysis indicated that water content exhibited a similar pattern to fresh matter content, showing no significant variation among the main factors of variety and inoculation methods or their interaction. Only the inoculation treatment (TriMix) demonstrated a significant effect (p < 0.001) on water content (**Supplementary Table 2**). Water content increased significantly (p ≤ 0.05) with increasing disease severity up to 31%. Control cobs and cobs with disease severity of 0% and 1-30% contained between 19.8% and 22.2% water (**Figure 3**).

### 3.5 Alpha-amylase activity

**Figure 4:**
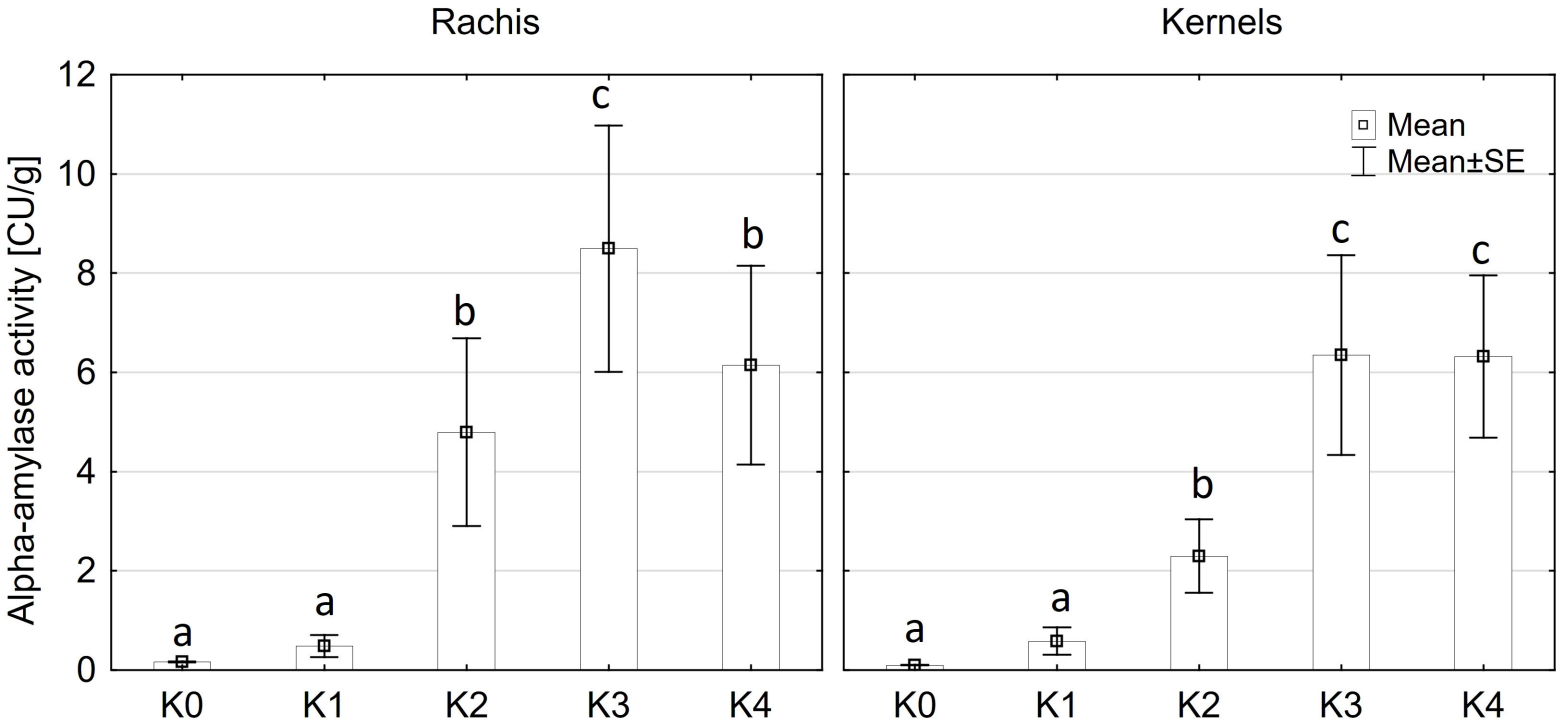
Mean alpha-amylase activity [CU/g] in rachis (left) and kernels (right) after artificial inoculation with *T. afroharzianum* at disease severity classes K0 (control), K1 (0% disease severity), K2 (1-30% disease severity), K3 (31-70% disease severity), and K4 (71-100% disease severity). Error bars represent standard error. Different letters show significant differences between classes (p ≤ 0.05, Tukey-Test). n = 8.

**Figure 5:**
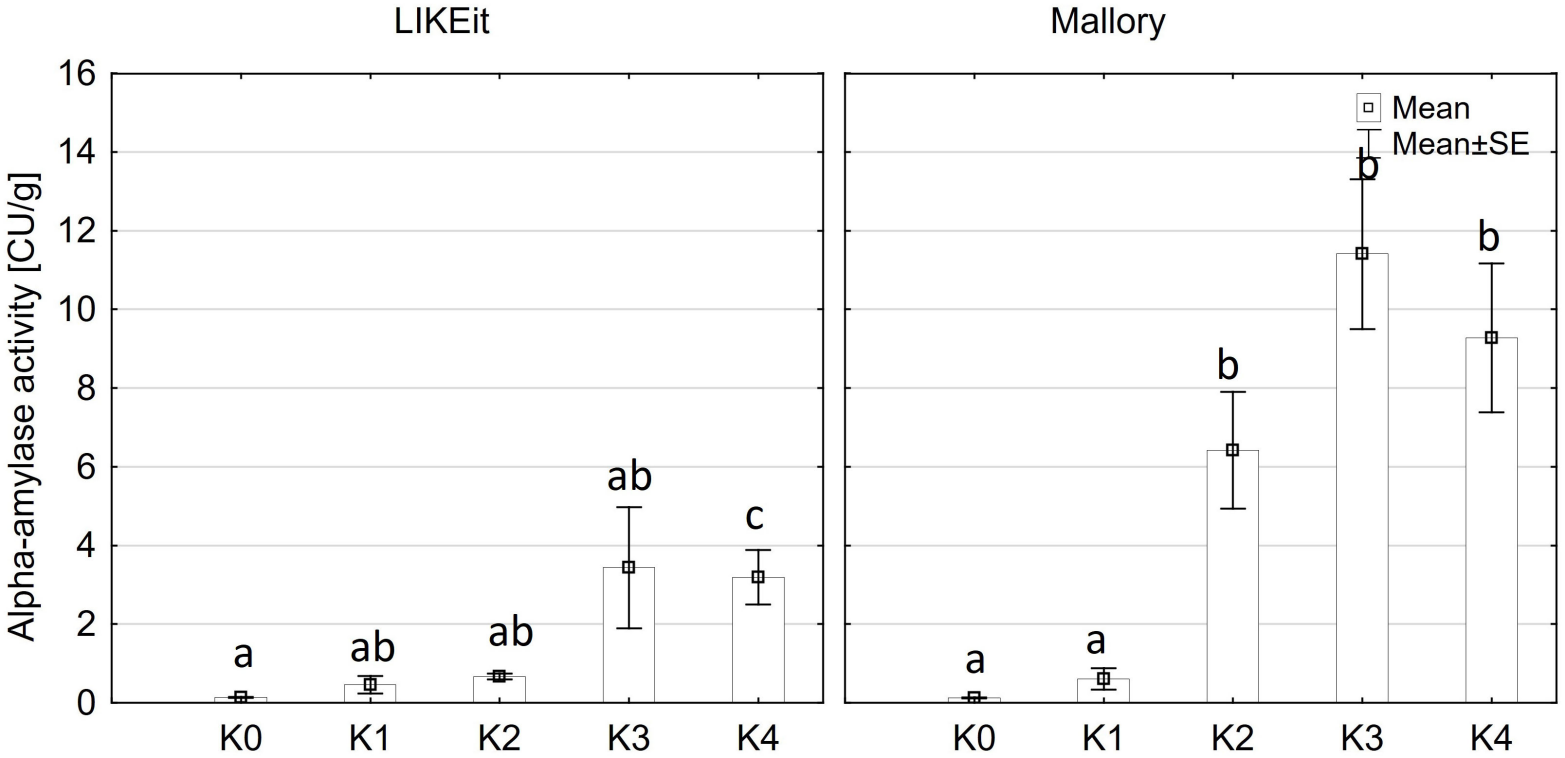
Mean alpha-amylase activity [CU/g] in the maize cob of LIKEit (left) and Mallory (right) after artificial inoculation with *T. afroharzianum* at disease severity classes K0 (control), K1 (0% disease severity), K2 (1-30% disease severity), K3 (31-70% disease severity), and K4 (71-100% disease severity). Error bars represent standard error. Different letters show significant differences between classes (p ≤ 0.05, Tukey-Test). n = 8.

Alpha-amylase activity exhibited significant differences among the two main factors variety and disease severity classes and by their interaction (p ≤ 0.001). Cob material did not display a significant effect on alpha-amylase activity, nor did any of the interactions involving cob material (**Supplementary Table 3**). Across both kernel and rachis materials, alpha-amylase activity demonstrated a notable increase with increasing disease severity, particularly within the 1-30% severity range (K2) (**Figure 4**), reaching its peak activity at 31-70% infection (8.4 CU/g). Subsequently, alpha-amylase activity continued to rise with increasing disease severity up to K3 (31-70%) across both varieties before declining in the highest disease severity class (K4) (**Figure 5**). Furthermore, Mallory exhibited significantly higher alpha-amylase activity compared to LIKEit. Specifically, the peak activity was recorded in LIKEit at K3 with 3.4 CU/g, while Mallory displayed its highest activity of 11.8 CU/g at 31-70% disease severity (K3).

### 3.6 Glucose content

**Figure 6:**
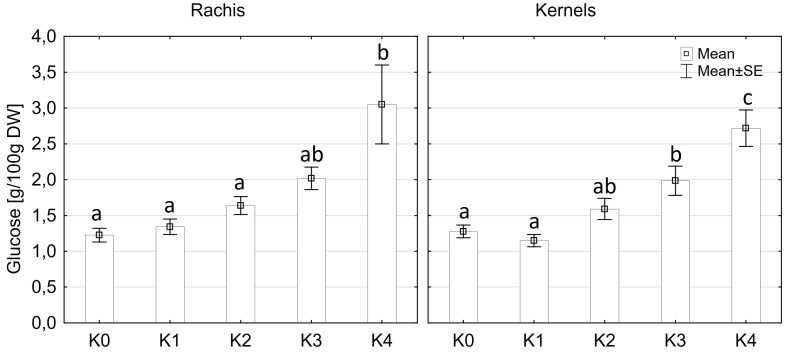
Mean glucose content [g/100g DW] in maize rachis (left) and kernels (right) after artificial inoculation with *T. afroharzianum* at disease severity classes K0 (control), K1 (0% disease severity), K2 (1-30% disease severity), K3 (31-70% disease severity), and K4 (71-100% disease severity). Error bars represent standard error. Different letters show significant differences between classes (p ≤ 0.05, Tukey-Test). DW, dry weight; n = 8.

**Figure 7:**
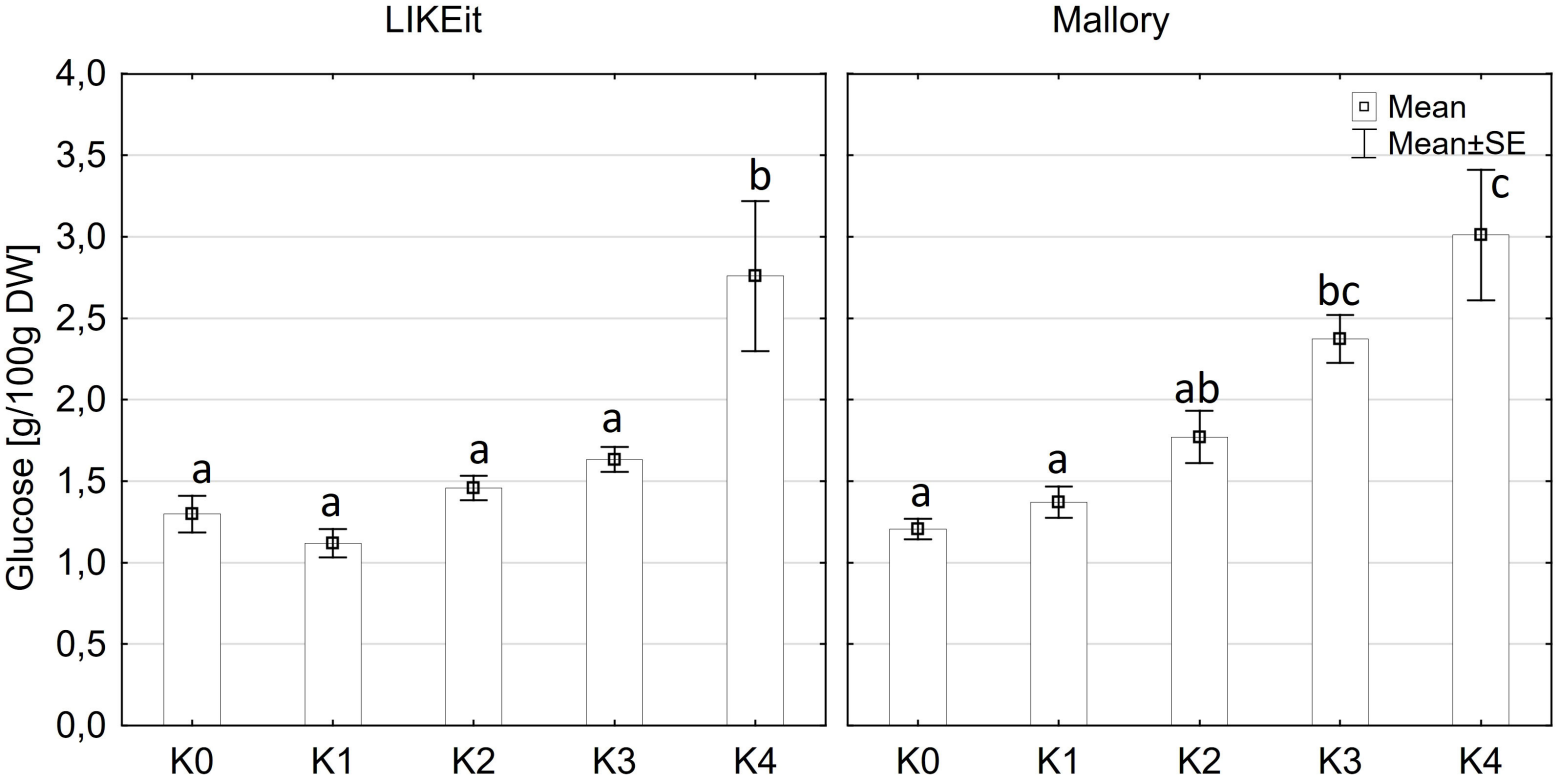
Mean glucose content [g/100 g DW] in maize cobs from LIKEit (left) and Mallory (right) after artificial inoculation with *T. afroharzianum* at disease severity classes K0 (control), K1 (0% disease severity), K2 (1-30% disease severity), K3 (31-70% disease severity), and K4 (71-100% disease severity). Error bars represent standard error. Different letters show significant differences between classes (p ≤ 0.05, Tukey-Test). n = 8.

Glucose content exhibited significantly variations among cultivars (p ≤ 0.05) and disease severity classes (p ≤ 0.001). However, there were no significant differences observed between cob material (kernels and rachis) or the interaction of all factors (**Supplementary Table 3**). Glucose content in both kernels and rachis increased with higher disease severity (**Figure 6**). Notably, no significant differences (p ≤ 0.05) in glucose content were detected between the control (K0), K1, and K2 classes, with mean contents ranging from 1.2 g to 1.6 g glucose/100 g DW (**Figure 6**). Glucose content showed an increasing trend with higher disease severity, reaching 2.0 g in class K3 and 2.8-3.1g in class K4. LIKEit exhibited a significantly (p ≤ 0.05) lower mean glucose content of 1.65 g/100 g DW than Mallory (2.23 g/100g DW). Interestingly, glucose content in LIKEit did not increase until the highest disease severity class K4, whereas Mallory showed a slight increase with disease severity, reaching its highest glucose content of 3.0 g/100 g DW in K4 (**Figure 7**).

### 3.7 C/N ratio

**Figure 8:**
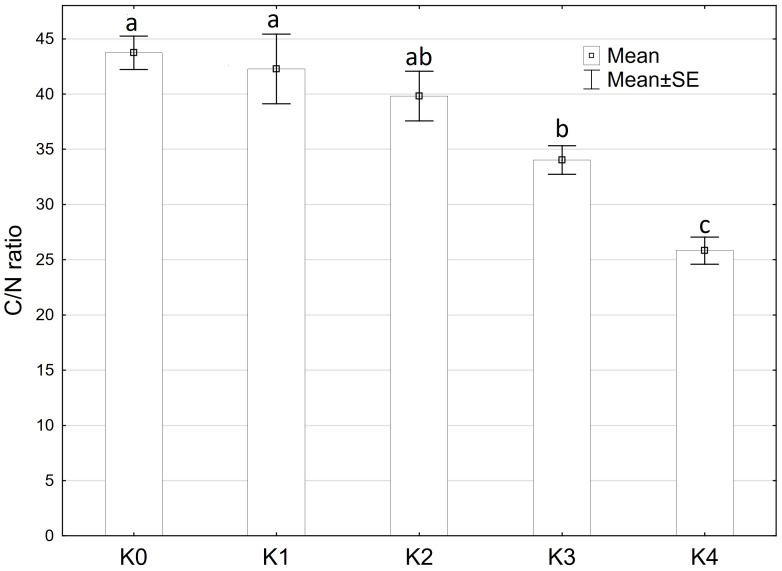
Mean C/N ratio of maize cobs after artificial inoculation with *T. afroharzianum* at disease severity classes K0 (control), K1 (0% disease severity), K2 (1-30% disease severity), K3 (31-70% disease severity), and K4 (71-100% disease severity). Error bars represent standard error. Different letters show significant differences between classes (p ≤ 0.05, Tukey-Test). n = 16.

The C/N ratio showed significantly (p ≤ 0.05) variations influenced by disease severity classes. Moreover, a highly significant difference (p ≤ 0.001) was observed in the C/N ratio concerning cob material, variety, and the interaction of cob material and disease severity classes. However, there were no significant differences detected between the interaction of cob material and variety, as well as interaction of all factors (**Supplementary Table 3**). The C/N ratio decreased significantly (p ≤ 0.05) with increasing disease severity. Control plants inoculated with water exhibited a mean N content of 1.0% and a C content of 45.3%. Both N and C percentages increased with rising disease severity. At the highest disease severity level (K4), maize cobs contained 1.8% N and 45.5% C, resulting in the lowest C/N ratio of 25.8% (**Figure 8**). No significant differences were observed between variety and plant material.

### 3.8 Germination rate and seedlings length

**Figure 9:**
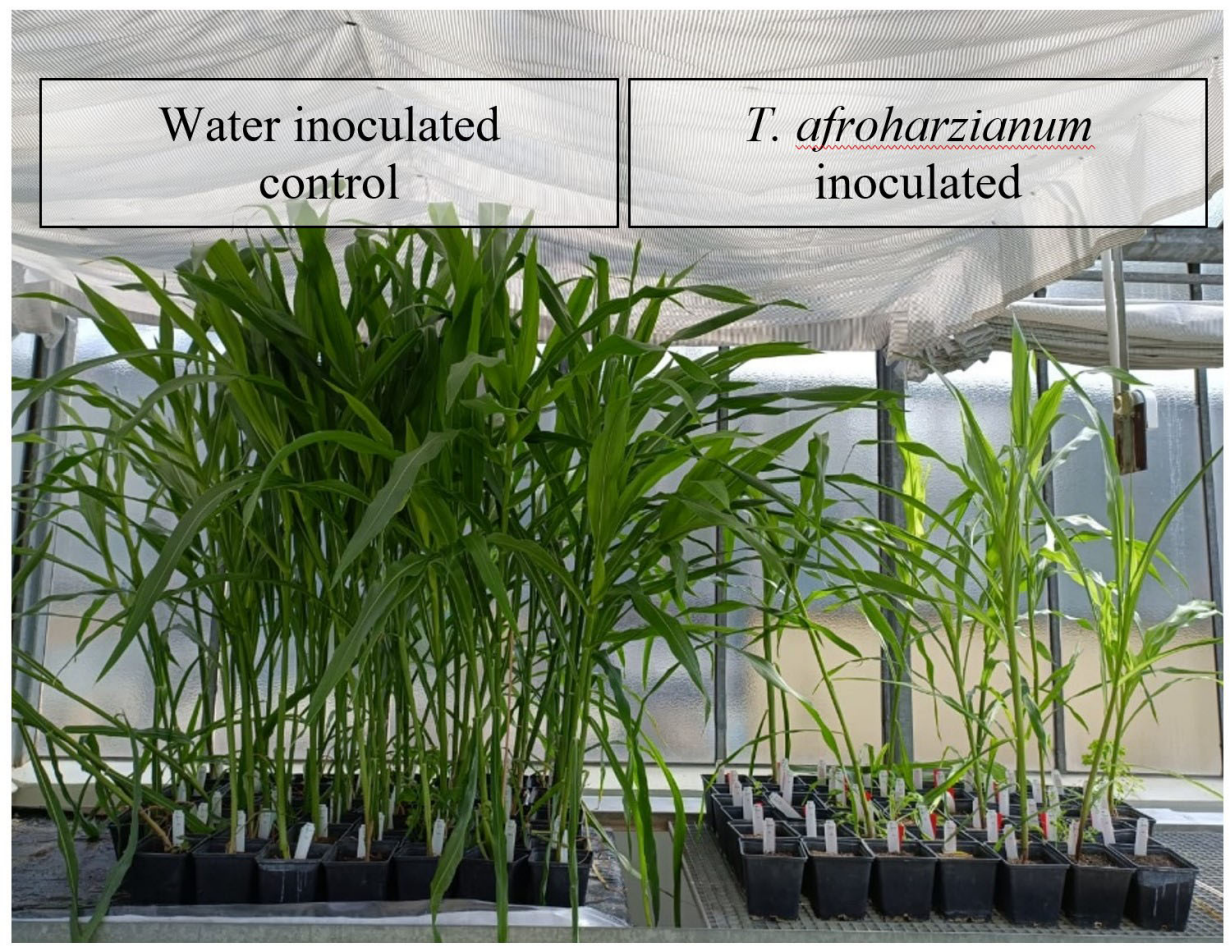
Germination rate and growth of seedlings after cob inoculation with water (left) and *T. afroharzianum* (right).

Significant differences (p ≤ 0.001) were observed in both germination rate and seedling length between *T. afroharzianum* inoculated and water-inoculated kernels. Germination was notably lower in *T. afroharzianum* infected kernels (24%) compared to control kernels inoculated with water (98%). Additionally, seedlings originating from infected kernels exhibited a significant reduction in plant height, measuring 133.3 cm, and displayed deformed growth compared to seedlings inoculated with water, which reached a height of 151.8 cm (**Figure 9**).

## 4 Discussion

The results of the semi-field trials indicate that *T. afroharzianum* is capable of infecting maize cobs under field conditions. The observed effects on fresh matter content and quality parameters highlight the significance of *Trichoderma* ear rot in maize crops.

Signs of *Trichoderma* ear rot have been described in previous publications as white mycelium growing between the kernels and on the husk leaves, with massive production of green to gray-green conidia. The symptoms occurred from the base to the middle part of the cob, covering all kernels and all layers of husk leaves. In addition, the infected cobs showed premature ripening of the kernels (Vincelli, 2014; Munkvold and White, 2016; Pfordt et al., 2020). Similar results were obtained after artificial inoculation under semi-field conditions. Severe symptoms of *Trichoderma* ear rot up to 100% were observed, including mycelial growth and extensive production of gray-green spores on the ear and husk leaves. In addition, the characteristic premature germination of kernels on the cob was observed.

The climate conditions during the semi-field trials appeared to be favorable at time point of flowering for *T. afroharzianum* infection. Mean temperature during the vegetation period in Goettingen was +2°C to +2.3°C above the long-term average, while precipitation was well below the long-term average (1971-2020). This is consistent with previous observations showing that *T. afroharzianum* naturally occurs more frequently after dry periods with high temperatures (Pfordt et al., 2020). Climate chamber experiments confirm this observation that growth rate (*in vitro*) and disease severity (*in vivo*) of *T. afroharzianum* increased at 25-35°C (data not shown). Furthermore, abiotic stresses such as drought or heat can weaken the plant’s natural defense mechanisms, making them more susceptible to infection by pathogens such as *T. afroharzianum* (Parsons and Munkvold, 2010; Pandey et al., 2015). With rising temperatures and changing weather patterns, *T. afroharzianum* infections are expected to increase.

Artificial inoculation was performed using two different methods, silk channel inoculation and needle pin inoculation, which represent two commonly discussed resistance types. These methods correspond to two commonly discussed types of resistance, namely silk channel resistance and kernel resistance, which are associated with specific aspects of plant defense against ear rot pathogens (Mesterházy et al., 2012). Needle pin inoculation is used to wound the tissue and observe fungal penetration and spread through kernel wounds, whereas silk channel inoculation showed the initial ability of the pathogen to penetrate the tissue without prior wounding (Reid, 1993; Reid and Hamilton, 1996; Chungu et al., 1996b; Clements et al., 2003). Kernel resistance refers to the ability of the plant to inhibit or limit the colonization and growth of ear rot pathogens in developing kernels, while silk channel resistance refers to the ability of the plant to prevent or limit the entry of ear rot pathogens through the silk channels (Windham and Williams, 2007). These resistance types represent different aspects of plant defense against ear rot pathogens and are related to specific infection sites (Mesterházy et al., 2012).

In this study, needle pin inoculation resulted in higher disease severity (60%) compared to silk channel inoculation (39%). This observation is consistent with previous findings for other ear rot pathogens, such as *Fusarium* spp. or *Aspergillus* spp., which indicate that wounding inoculation methods result in higher infection rates compared to non-wounding methods (Chungu et al., 1996a; Clements et al., 2003; Windham and Williams, 2007). When plant tissue is wounded during inoculation, it creates entry points for the pathogens, facilitating their colonization and establishment within the plant. The wounds provide easier access route, bypassing the plant’s initial defense barriers for the pathogens to invade the plant tissues, increasing the likelihood of infection and subsequent disease development (Chungu et al., 1996b). This makes needle pin inoculation a reliable inoculation method for artificial *T. afroharzianum* infection in the field, resulting in homogenous disease severity and consistent disease outcomes. Silk channel inoculation also resulted in high disease severity of individual cobs, but the infection rate was highly variable between cobs of the same treatment, resulting in a high standard error. This can be attributed to several factors. First, the success of silk channel inoculation depends on the age of the silk channels. Older silk channels may be less susceptible to infection than younger ones (Reid et al., 1992a). Second, environmental conditions play an important role in the effectiveness of silk channel inoculation. Factors such as humidity, temperature, and pathogen load in the spore suspension can influence the establishment and spread of pathogens through the silk channels (Reid et al., 1992b, 1995). This variability makes it difficult to obtain consistent and reproducible results using silk channel inoculation. However, silk channel inoculation better mimics natural infection in the field as there are no visible symptoms of insect or birds injury. This suggests that infection is either from infected silk channels or from systemic growth within the plant.

Mallory had a higher disease severity (72%) than LIKEit (36%), indicating a different susceptibility to *Trichoderma* ear rot infection. These findings indicate that different maize genotypes may differ in their susceptibility to *T. afroharzianum*. Mallory appears to be more susceptible to infection, leading to a higher infection rate, while LIKEit has a lower susceptibility, indicating some level of resistance or tolerance to the pathogen. *Trichoderma* ear rot infection resulted in significant changes in the fresh matter content of the cobs. Affected cobs appeared smaller and lighter, with soft and watery kernels and husk leaves adhering to the cob. Fresh matter content decreased significantly with increasing disease severity. High infection rates with disease severity above 70% resulted in a significant reduction in fresh matter content of up to 55% compared to control plants. *T. afroharzianum* interferes with the normal kernel development and maturation, resulting in reduced size and weight compared to healthy cobs. Surprisingly, even at 0% visible disease severity, the fresh matter content is significantly reduced. This suggests that *T. afroharzianum* can affect plants at a physiological level, disrupting their growth and development in the absence of visible symptoms. Such symptomless infections can nevertheless have a significant impact on the fresh matter content and quality of maize crops.

In addition, the results suggest that *T. afroharzianum* infection affects seedling germination and growth. The germination rate of previously infected kernels was significantly reduced compared to water-inoculated cobs without symptoms. Only 22% of the kernels were able to germinate and showed stunted and deformed growth compared to control plants. The presence of the pathogen on the kernels may inhibit or impair the germination process, leading to reduced germination rates and interfering with normal seedling development. The presence of the pathogen in seeds can lead to lower germination rates and poorer seedling vigor, further impacting crop yields (MacGee, 1998). These effects can contribute to food insecurity, especially in regions heavily reliant on maize as a staple food. Effective disease management strategies are crucial to mitigate these impacts and ensure sustainable maize production (Agarwal and Sinclair, 2014).

**Figure 10:**
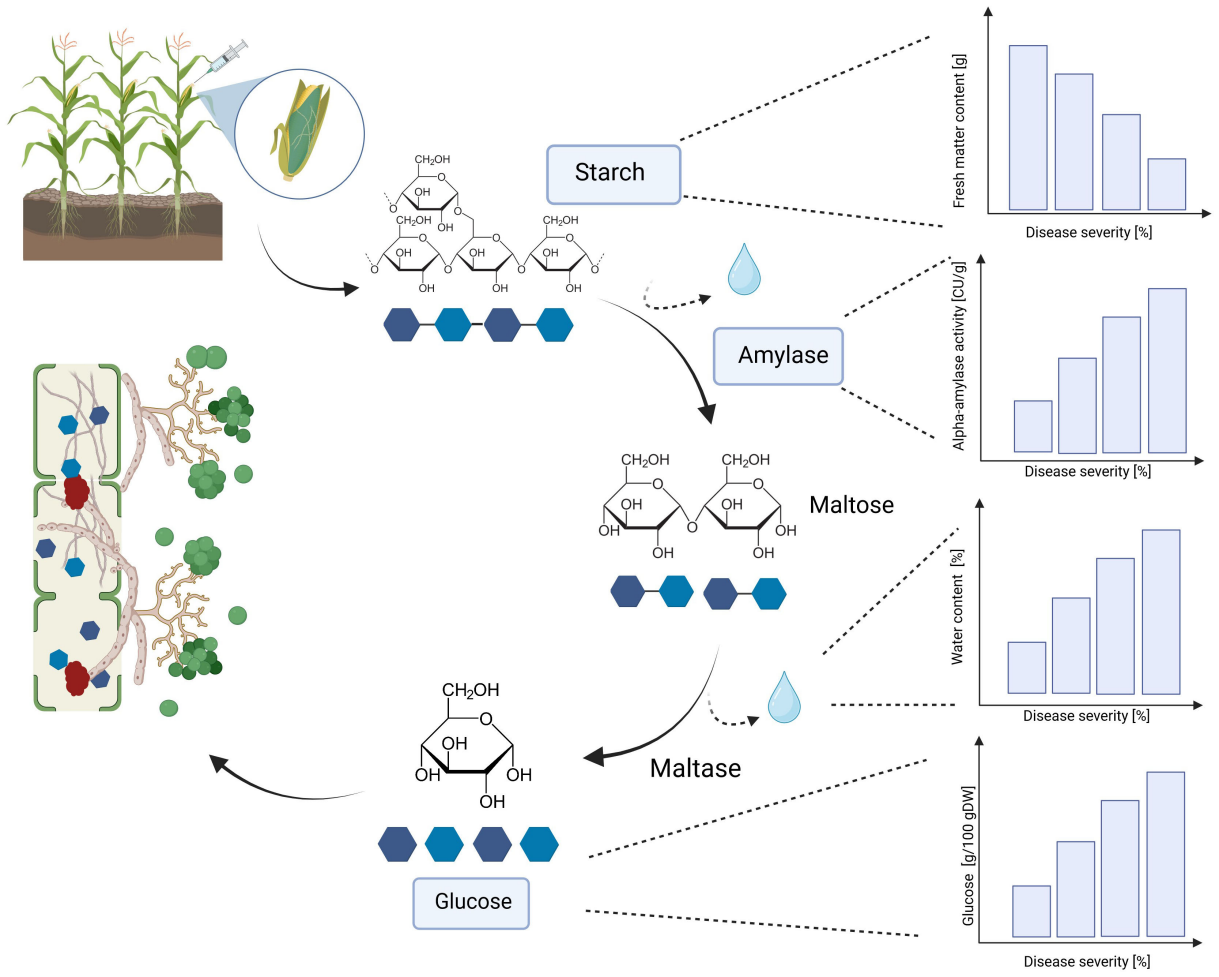
Effect of *T. afroharzianum* infection on fresh matter content, alpha-amylase activity, water content, and glucose content in maize cobs. DW, dry weight (Created with BioRender.com).

A significant increase in alpha-amylase activity with increasing disease severity was observed in this study. Water-inoculated control plants had a low alpha-amylase activity of 0.3 CU/g DW, which increased significantly to 11.5 CU/g DW at 30-70% disease severity in Mallory. Along with alpha-amylase activity, water content also increased with increasing infection levels. Low-infected cobs and water-inoculated control cobs generally contained about 20% water, but water content increased up to 30% in highly infected plants. The results of this study also showed a significant increase in glucose content, which increased from 1.2 g/DW to 3.1 g/DW at the highest infection level of 70%. The significant increase in alpha-amylase activity with higher disease severity indicates that *T. afroharzianum* infection triggers the breakdown of starch into simpler sugars, such as glucose. This enzymatic activity reduces starch content and increases glucose levels, affecting the nutritional and processing quality of the maize. The significant differences in glucose content between LIKEit and Mallory can also be attributed to genetic differences in their response to *T. afroharzianum* infection. Mallory’s higher glucose content indicates a higher susceptibility to the pathogen, leading to more extensive starch degradation. In contrast, LIKEit’s lower glucose content suggests a better ability to tolerate the infection, resulting in less starch degradation. These insights highlight the role of pathogen-induced enzymatic changes in altering maize kernel physiology and quality. Fungi are heterotrophic organisms that must obtain food by absorbing nutrients from organic matter in their environment. Most fungi, including *Trichoderma* spp., obtain their food by decomposing dead or decaying organic matter such as starch, cellulose, and lignin (de Azevedo et al., 2000; Agrios, 2008). They secrete enzymes, including amylase, to break down complex organic compounds into simpler forms that can be absorbed and utilized as nutrients (Webster and Weber, 2012). Alpha-amylase is an enzyme that plays a crucial role in the breakdown of complex carbohydrates, such as starch, into simpler sugars (Niu et al., 2023). It is produced by various organisms, including humans, animals, plants, and fungi, to break down the alpha-1,4-glycosidic bonds between glucose units in starch (Dziedzic, 2012). During the hydrolysis of the alpha-1,4-glycosidic bonds by amylase, water molecules are released as a byproduct. The hydrolysis of each bond releases a glucose molecule along with a water molecule. Glucose is a monosaccharide that plays an important role in the energy metabolism of organisms (Jennings, 1995). The higher the amylase activity, the more starch is degraded and the higher the glucose content (**Figures 6**, **7**). It may be suggested by the results of the studies that *T. afroharzianum* itself releases alpha-amylase enzymes into the infected plant tissue to break down the starch into simpler sugars that are more easily absorbed and utilized by the fungi. This enzymatic breakdown of starch into glucose provides *T. afroharzianum* with an available source of energy and nutrients that supports its colonization and survival within the plant (Abdul et al., 2020). The degradation of starch to glucose by *T. afroharzianum* may contribute to the decrease in dry matter content of infected maize cobs. Starch is a major component of maize kernels (60-75% of DW) and accounts for a significant portion of their DW (Lütke Entrup et al., 2013). As the starch is degraded into glucose, the glucose concentration in the infected plant tissue increases. This allows the fungus to access the energy stored in the starch and use it for its own growth and development. Each enzymatic step releases a byproduct of the hydrolysis reaction, contributing to the overall observed increase in water content in infected plant tissue. These findings further explain the soft rotting symptoms of the maize cob (**Figure 10**). The results of the study indicate a connection between alpha-amylase activity, glucose content, water content, and overall disease severity in the different maize varieties. The higher disease severity observed in Mallory resulted in higher alpha-amylase activity and glucose content, indicating more active enzymatic degradation of starch within the infected tissue. In contrast, the LIKEit cultivar showed lower degree of disease severity, indicating lower susceptibility to *Trichoderma* ear rot infection. Consequently, alpha-amylase activity and glucose content were also lower in this cultivar compared to Mallory. This suggests that the LIKEit cultivar may have inherent mechanisms or traits that confer resistance or reduced susceptibility to *Trichoderma* ear rot infection, resulting in lower alpha-amylase activity and glucose content.

Alpha-amylase production by *T. afroharzianum* can disrupt normal physiological processes in maize kernels, resulting in reduced starch content, higher water content and altered nutrient composition. As a result, the biomass and weight of infected cobs are reduced, and overall quality is compromised. This can affect the processing and storage characteristics of infected maize cobs and lead to difficulties in handling, preservation, and utilization. Increased water content in the cobs affects harvest timing and storage. Increased moisture content in the crop may delay the optimum harvest time or increase the cost of drying to reduce the water content to an appropriate level for storage. Otherwise, insufficiently dried crops can lead to further problems during storage, such as mold growth, spoilage, and shortened shelf life.

Premature germination is a common symptom observed in maize kernels infected with *Trichoderma* ear rot. *Trichoderma* infection appears to disrupt the normal dormancy of the seed, leading to premature activation of metabolic processes associated with germination (Thévenot et al., 1992; Subbarao et al., 1998). In healthy maize kernels, the seed remains dormant until biochemical reactions are triggered and enzymes such as alpha-amylase are activated to stimulate embryo growth (Shaik et al., 2014; Patwa and Penning, 2020). Alpha-amylase is naturally produced in the aleurone layer of the endosperm of maize kernels to initiate the degradation of starch into simpler sugars. These sugars can then be utilized by the developing embryo as an energy source for germination (Nelson and Pan, 1995). However, *T. afroharzianum* appears to interrupt this dormancy by producing alpha-amylase, which triggers the germination process even when the seed is still inside the husk leaves. This can be observed visually by the presence of shoot growth on the cobs. Premature germination can also be caused by other fungi, such as *Fusarium* spp., through the production of the hormone gibberellin (Vrabka et al., 2018). Alpha-amylase production by *T. afroharzianum* can disrupt normal physiological processes within the maize kernel, resulting in changes in the timing and sequence of germination (Thévenot et al., 1992). Premature germination caused by *Trichoderma* ear rot is considered detrimental to seed viability and overall plant development. It can result in weakened seedlings and reduced seed quality due to premature depletion of energy reserves stored in the seed. In addition, the emergence of the embryonic plant within the husk leaves makes the cob more susceptible to further infection.

With increasing disease severity, there was a notable significant decrease in the C/N ratio. The C-to-N (C/N) ratio is a metric that compares the relative amounts of C and N present in a substance such as organic matter or plant tissue (Anderson and Domsch, 1989). In general, a higher C/N ratio indicates a higher proportion of C relative to N, implying that the organic matter is relatively C-rich (Amelung et al., 2018). Fungi such as *Trichoderma* spp. utilize organic matter by actively degrading C-rich compounds such as lignin or starch as a nutrient source (Liming and Xueliang, 2004). As a result, the degradation of these compounds by fungi can lead to a decrease in the C/N ratio of the material as C is released into the environment in the form of CO_2_ and other simple C compounds. In addition, fungi can also take up N from the environment and incorporate it into their own biomass. This can further increase the N content of the organic material and decrease the C/N ratio (Fontaine et al., 2003; Abro et al., 2011). The decrease in the C/N ratio is primarily due to the release of C during the degradation of starch by *T. afroharzianum*. During the degradation of starch, the C present in the starch is released into the environment as CO_2_ and glucose. In addition, *T. afroharzianum* utilizes N for its growth and metabolic activity, which contributes to a further increase in the total N content and subsequently to a reduction in the C/N ratio.

In conclusion, *Trichoderma* ear rot in maize significantly affects grain quality and fresh matter content. It appears as severe mycelial growth on the kernels and outside of the husk leaves with massive production of gray-green spores and leads to a reduction in grain weight, decreased starch content, altered nutrient composition, and increased water content. The enzymatic activity of *T. afroharzianum*, particularly the production of alpha-amylase, contributes to the degradation of starch into simpler sugars such as glucose. In addition, *Trichoderma* ear rot negatively affects seedling development by causing premature germination, reduced germination rates, and abnormal growth. These effects have implications for agricultural food and feed production. The decrease in grain quality, including reduced starch content and altered nutrient composition, can affect both food and feed production. The increase in water content can also cause problems during storage, as higher moisture levels can lead to mold growth and spoilage. Therefore, it is important to monitor the incidence of *T. afroharzianum* infection in maize crops and to implement effective disease management strategies. Further research is needed to better understand the mechanisms of *T. afroharzianum* infection and its impact on the fresh matter content and quality parameters of maize. This research can contribute to the development of more effective management strategies and improve the sustainability of maize production.

## Data availability statement

The raw data supporting the conclusions of this article will be made available by the authors, without undue reservation.

## Author contributions

AP: Conceptualization, Validation, Writing – original draft, Data curation, Funding acquisition, Project administration, Supervision, Visualization. LS: Investigation, Methodology, Writing – review & editing. TR: Investigation, Methodology, Writing – review & editing. MN: Project administration, Supervision, Validation, Writing – review & editing.
