# Supplementary material for: Impact of Trichoderma afroharzianum infection on fresh matter content and grain quality in maize
Source: Front Plant Sci. 2024 Jul 23;15:1436201. doi: 10.3389/fpls.2024.1436201 (PMC11300376; doi:10.3389/fpls.2024.1436201)
Supplement: Supplementary file 1 [file DataSheet_1.docx]

Supplemental material

Suppl. Table 1. Utilized *T. afroharzianum* isolates to generate a composite spore suspension termed TriMix. F = France; G = Germany

| **Species** | **Isolate** | **Origin** | **Host** | **Year of isolation** |
| --- | --- | --- | --- | --- |
| *T. afroharzianum* | Tri1 | Croix de Pardie (F) | Maize | 2018 |
|  | Tri2 | Künzing (G) | Maize | 2018 |
|  | Tri3 | Pocking (G) | Maize | 2018 |

Suppl. Tab 2. Significance of the effect of isolate, inoculation method and variety on the disease severity of *T. afroharzianum*, fresh matter content and water content of maize cobs.

| **Factor** | **Disease severity** | **Fresh matter content** | **Water content** |
| --- | --- | --- | --- |
| Isolate | *** | *** | *** |
| Inoculation method (IM) | * | n.s. | n.s |
| Variety (V) | * | n.s. | n.s |
| IM x V | * | n.s. | n.s |

* p≤0.05; ** p≤0.01; *** p≤0.001; n.s not significant

Suppl. Tab. 3: Significance of the effect of disease severity classes, cob material and variety thus there interactions on amylase activity, glucose content and C/N ratio in maize cobs.

| **Factor** | **Amylase activity** | **Glucose content** | **C/N ratio** |
| --- | --- | --- | --- |
| Disease severity classes (DS) | *** | *** | * |
| Cob material (CM) | n.s | n.s | *** |
| Variety (V) | *** | * | *** |
| DS x V | *** | n.s | * |
| DS x CM | n.s | n.s | *** |
| CM x V | n.s | n.s | n.s |
| DS x CM x V | n.s | n.s | n.s |

* p≤0.05; ** p≤0.01; *** p≤0.001; n.s not significant


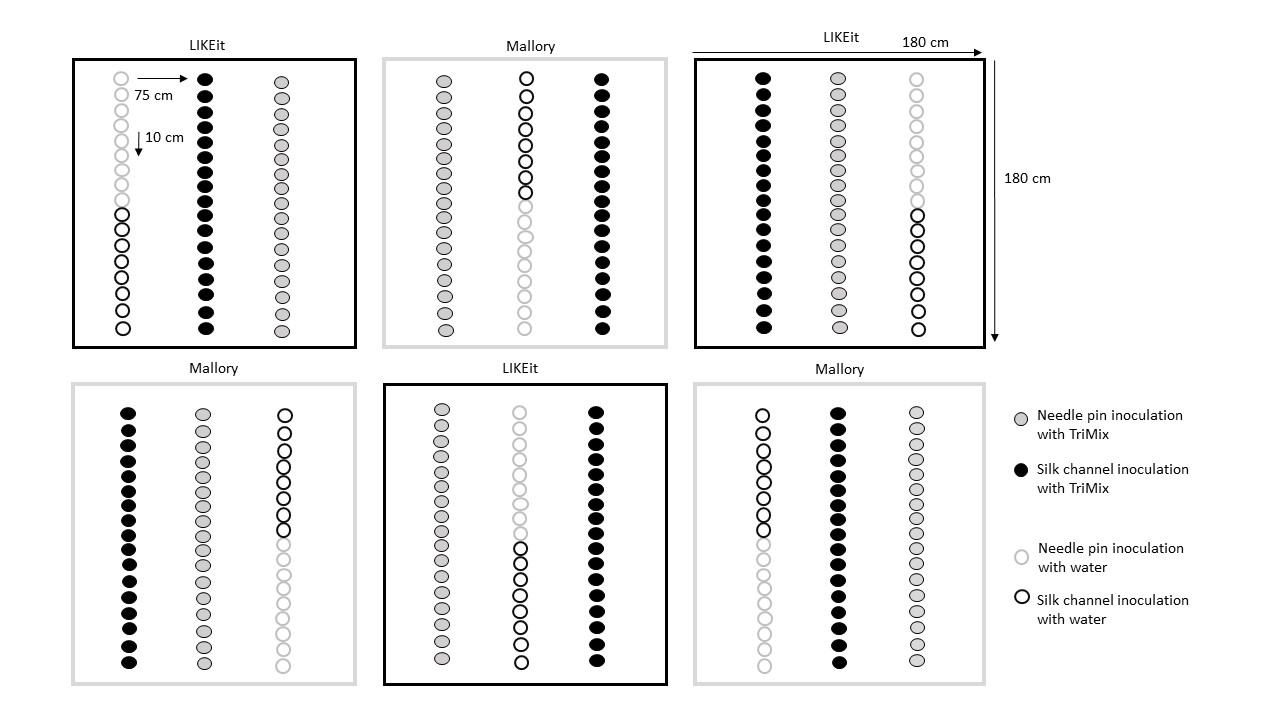


Suppl. Figure 1. Schematic representation of the experimental design showing the planting arrangement and plot design. Two of the three rows were inoculated with the pathogenic *T. afroharzianum* mix isolate (TriMix), one row inoculation with silk channel and the other by inoculation with the needle pin. The remaining row served as a control, with half of the plants inoculated with water through the silk channel and the other half by needle pin inoculation.
